# Supplementary figures and images for: Age-Related and Heteroplasmy-Related Variation in Human mtDNA Copy Number
Source: PLoS Genet. 2016 Mar 15;12(3):e1005939. doi: 10.1371/journal.pgen.1005939 (PMC4792396; doi:10.1371/journal.pgen.1005939)

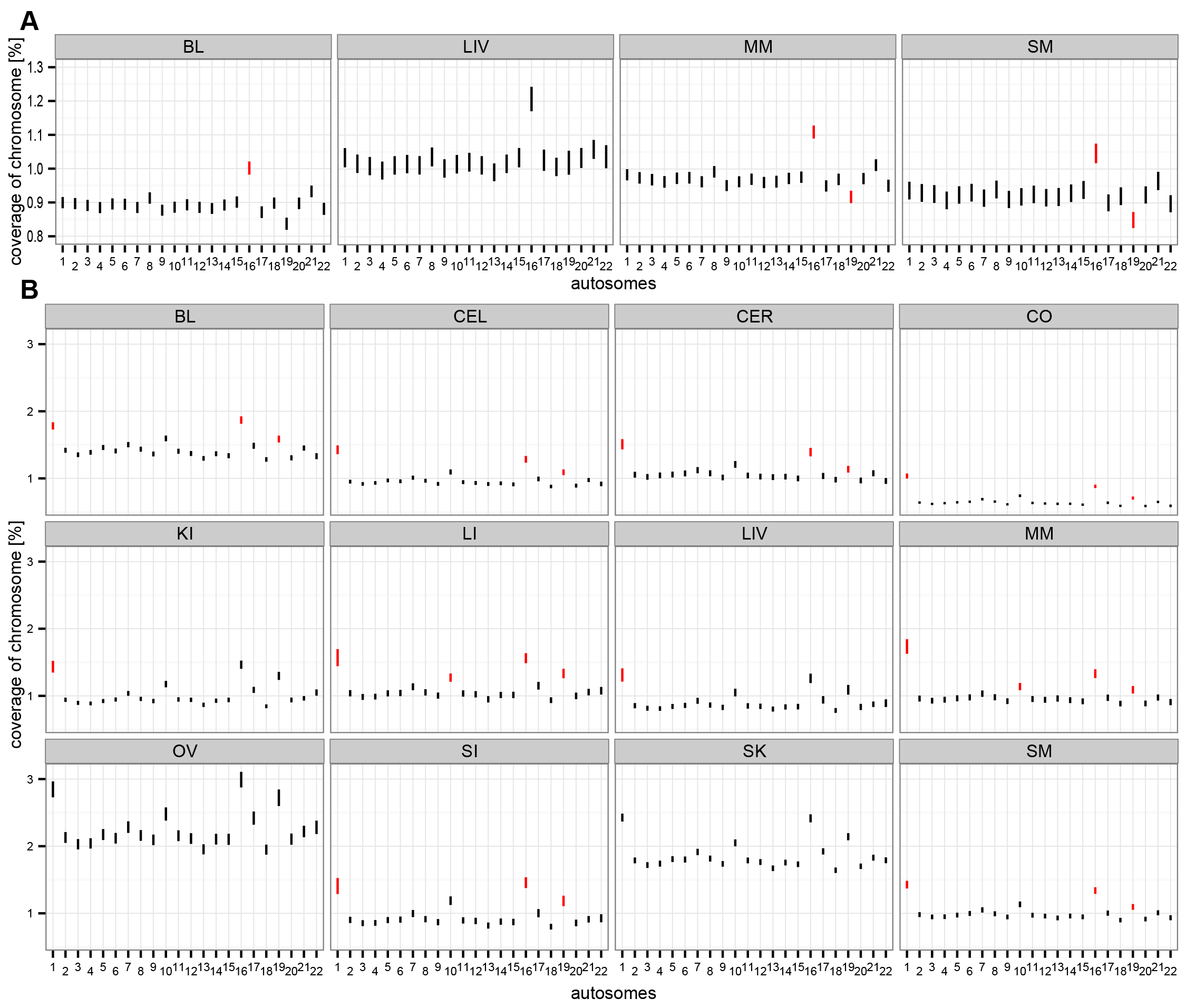

Supplement: S1 Fig — Each bar indicates the 95% confidence interval of the chromosomal coverage. Red bars indicate significant outliers according to SD. (TIF) [file pgen.1005939.s001.tif]

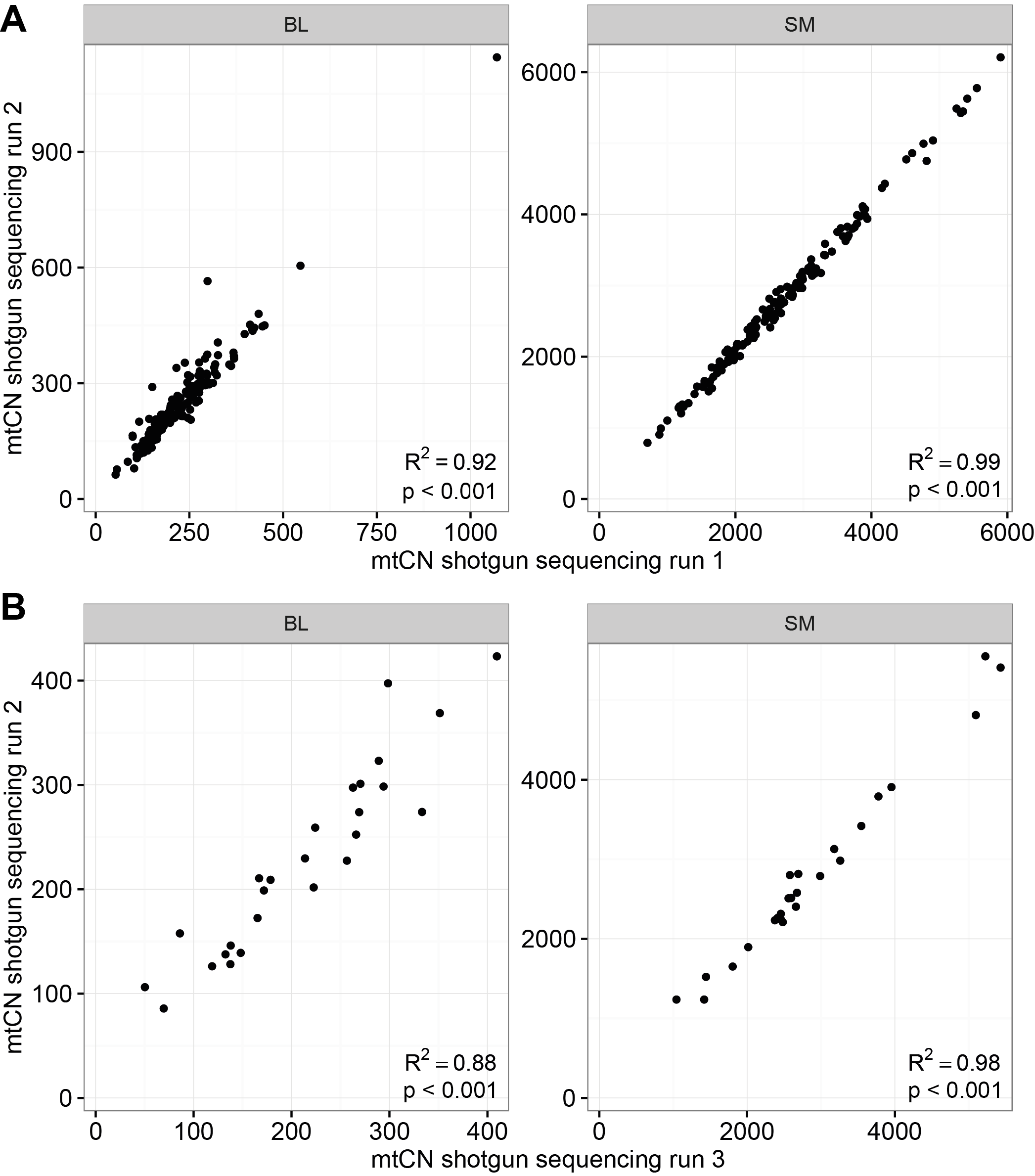

Supplement: S2 Fig — A BL and SM samples were sequenced in two independent sequencing runs of the same libraries. B independent libraries were prepared from a subset of 26 individuals for BL and SM samples and sequenced on a MiSeq, mtCNs from this sequencing run are plotted against the corresponding mtCNs from the first library. R2 represents the coefficient of determination of a linear regression analysis adjusted for the degrees of freedom. (TIF) [file pgen.1005939.s002.tif]

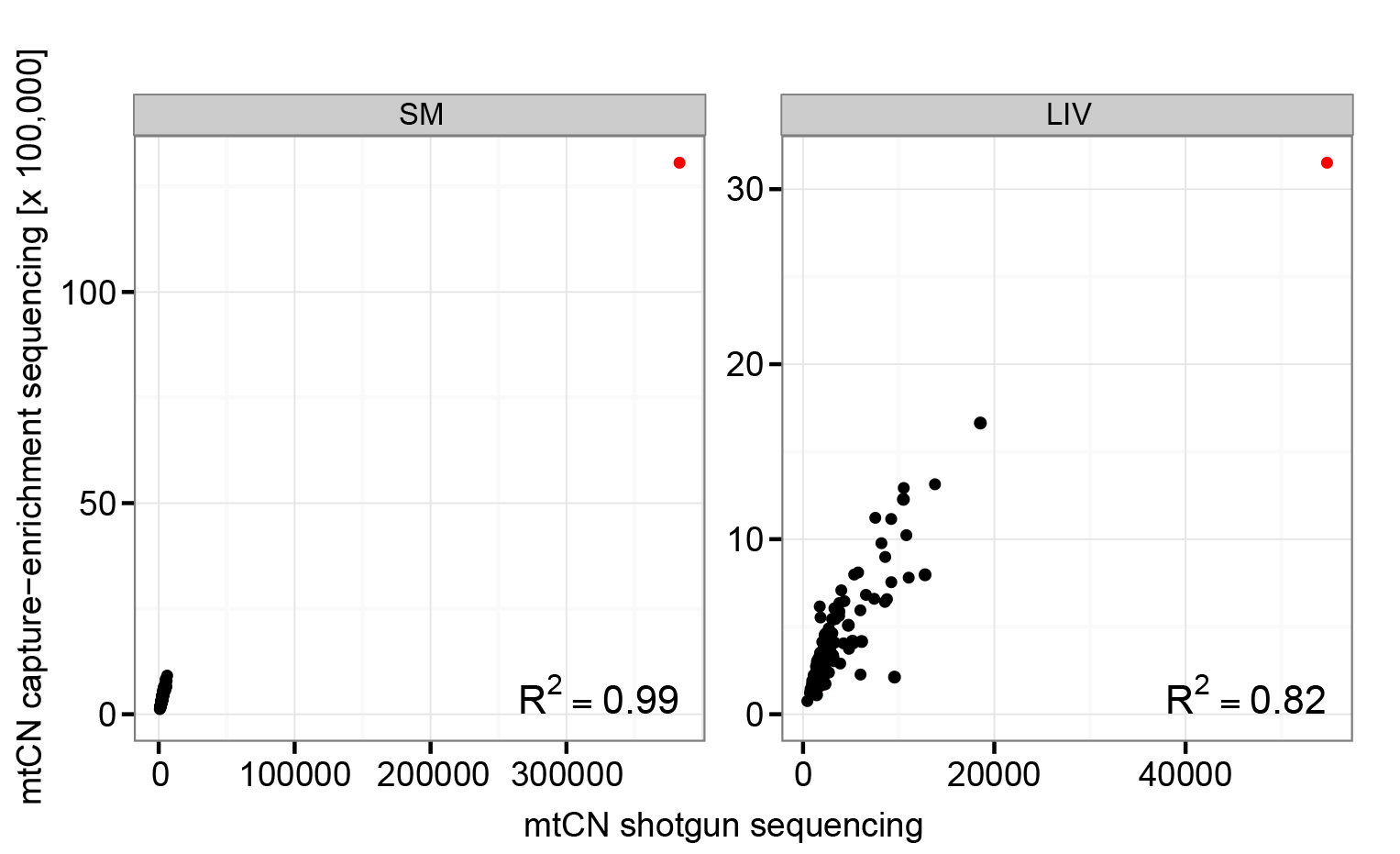

Supplement: S3 Fig — One SM sample and one LIV sample (red) had very high mtCNs, and were excluded from the correlation analyses for violating the assumption of a normal distribution. (TIF) [file pgen.1005939.s003.tif]

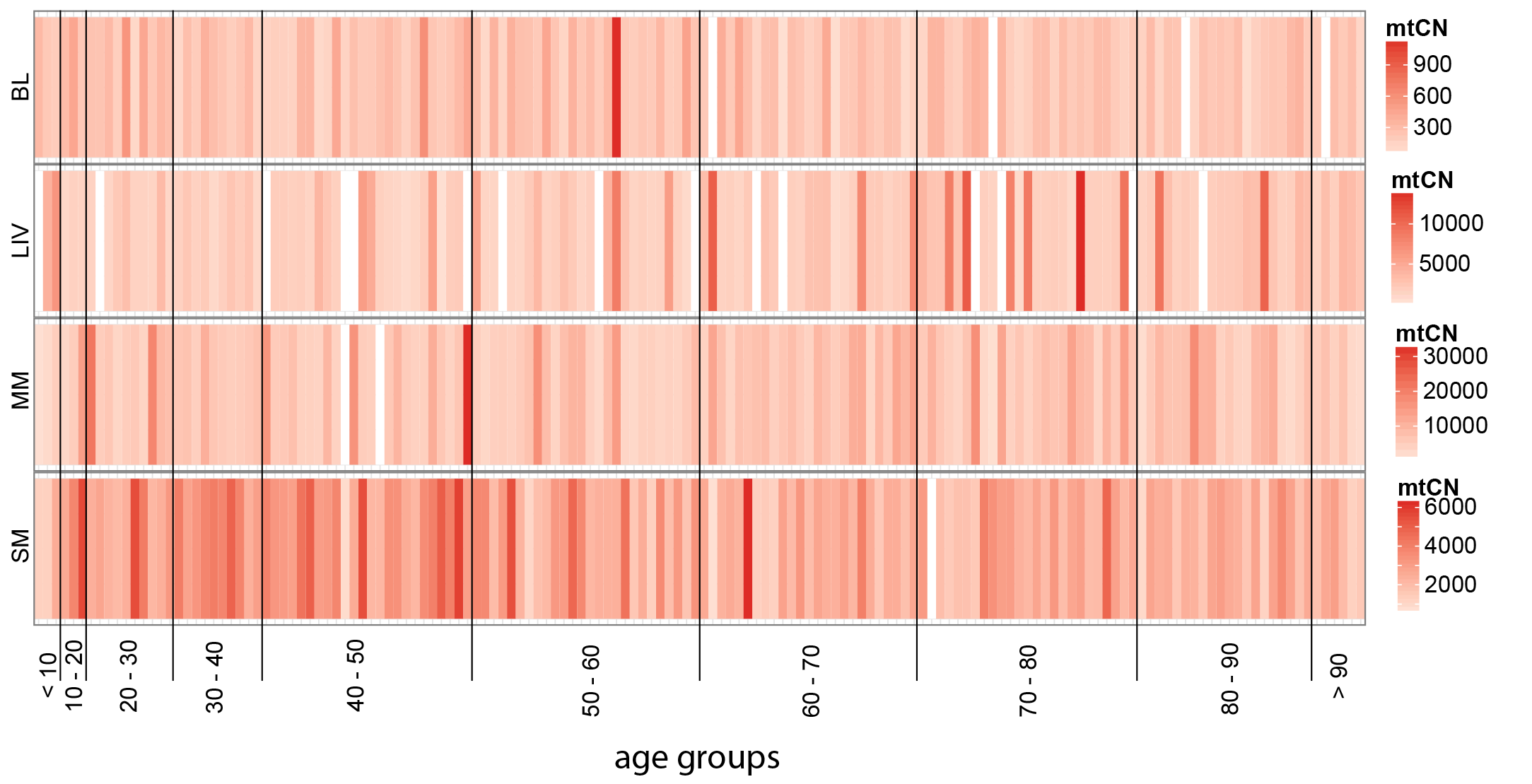

Supplement: S4 Fig — Vertical bars indicate single individuals, sorted from young (left) to old (right). Coloring of a vertical bar indicates the mtCN according to the scale on the right of each plot. (TIF) [file pgen.1005939.s004.tif]

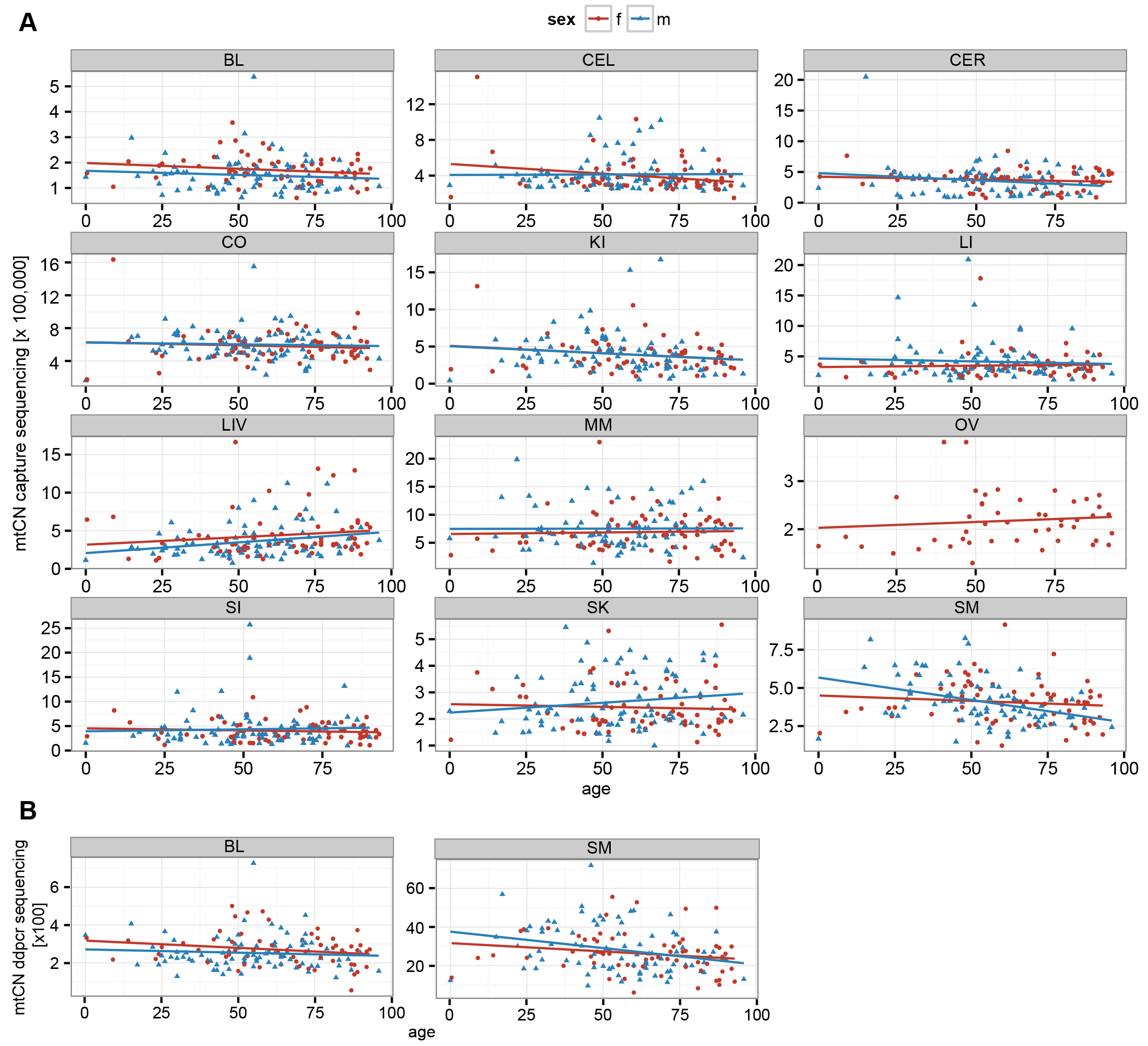

Supplement: S5 Fig — Males (m) and females (f) are distinguished. (A) capture-enrichment. (B) ddPCR. (TIF) [file pgen.1005939.s005.tif]

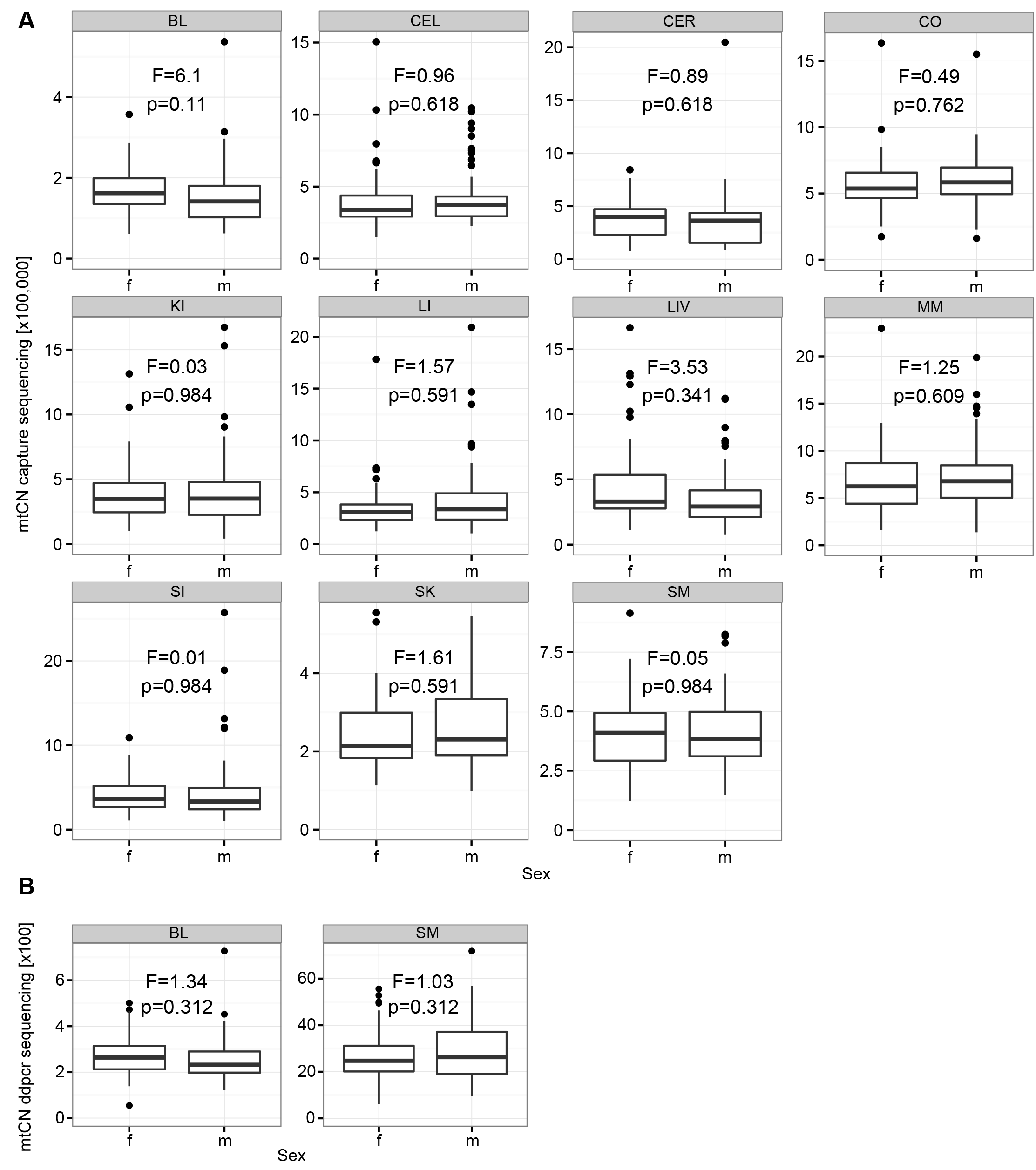

Supplement: S6 Fig — Males (m) and females (f) are indicated. F- and p-values are specified for each tissue. (A) capture-enrichment. (B) ddPCR. (TIF) [file pgen.1005939.s006.tif]

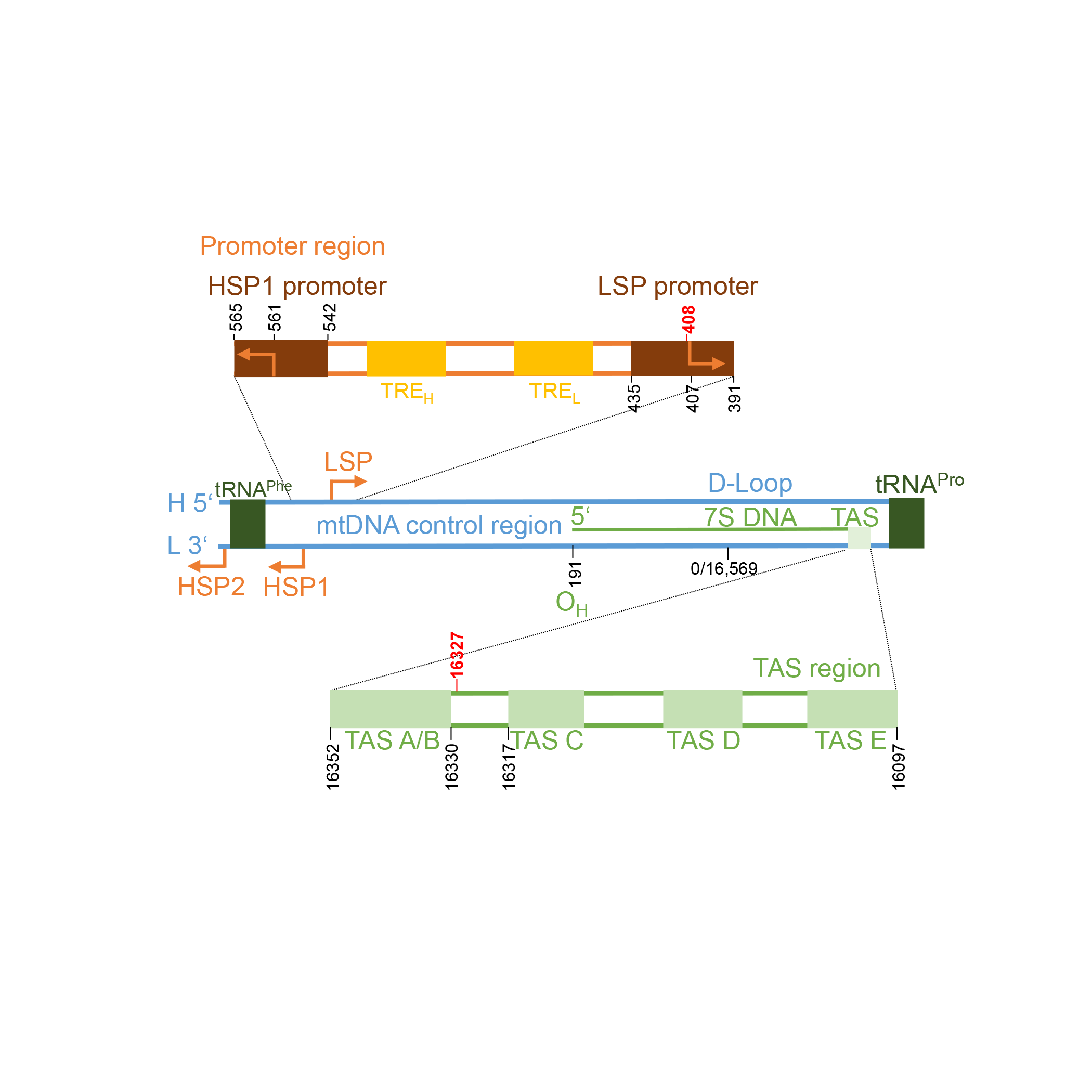

Supplement: S7 Fig — A short RNA primer is transcribed from the light strand promoter (LSP). Replication starts at OH (heavy strand origin of replication). Many replication events terminate in the TAS-region leading to release of a 7S DNA that stays attached to the D-loop region. Positions 408 and 16,327 are located within the LSP or TAS-region, respectively. (TIF) [file pgen.1005939.s007.tif]
